# The anesthesiologist’s perspective in choosing between robotic-assisted and conventional laparoscopic urological surgery: a propensity score-matched analysis

**DOI:** 10.1186/s12871-026-03730-1

**Published:** 2026-03-31

**Authors:** Meiyu Wei, Ce Zhang, Dongnan Hou, Lijie Wen, Bo Yang

**Affiliations:** 1 Department of Anesthesia, The Second Hospital of Dalian Medical University, Pu Wan branch, Dalian, Liaoning 116100 China; 2 Development Planning and Quality Management, The Second Hospital of Dalian Medical University, Dalian, 116000 China; 3 Department of Urology, The Second Hospital of Dalian Medical University, Dalian, Liaoning 116000 China

**Keywords:** Robotic-assisted laparoscopic surgery, Conventional laparoscopic surgery, Anesthesia, Decision-making, Urological surgery, Postoperative recovery

## Abstract

**Background:**

Comparative studies between robotic-assisted laparoscopic surgery (RALS) and conventional laparoscopic surgery (CLS) primarily focus on surgical outcomes, with limited emphasis on the anesthesiologist's perspective. This study aimed to compare anesthesia-related Enhanced Recovery After Surgery (ERAS) outcomes to inform surgical decision-making.

**Methods:**

This retrospective study included 831 patients who underwent partial nephrectomy or radical prostatectomy for renal or prostate cancer between September 2021 and October 2023. Propensity score matching (PSM) was performed in a 1:1 ratio based on age, gender, BMI, surgery type, and comorbidities. Primary outcomes included anesthesia time, surgical time, recovery time, and resting/activity VAS scores on postoperative day one (POD1). Secondary outcomes comprised ambulation status, nausea/vomiting, dizziness, and anxiety incidence. Safety indicators were pharyngeal pain, respiratory depression, and oxygen requirement.

**Results:**

After PSM, 252 patients were allocated to each group. The RALS group demonstrated significantly longer anesthesia and surgical times compared to the CLS group (*P* < 0.05). PACU recovery time showed no significant difference. While Rest-VAS on POD1 was similar, Act-VAS was significantly higher in the RALS group (*P*<0.05). The proportion of patients achieving autonomous mobilization (Grade 1) was significantly lower in the RALS group(*P*<0.01). Incidences of nausea/vomiting and dizziness were significantly higher in the RALS group (*P*<0.05). Anxiety incidence was significantly lower in the RALS group (*P*<0.05). No significant differences were found in sore throat, respiratory depression, or oxygen requirement rates.

**Conclusions:**

These findings suggest that from an anesthesiological perspective, RALS is associated with longer surgical and anesthesia time. The observed differences in postoperative recovery indicators highlight the importance of considering patient physiological reserve when selecting a surgical approach. However, given the observational nature of this study, these findings should be considered hypothesis-generating and warrant prospective validation.

**Supplementary Information:**

The online version contains supplementary material available at 10.1186/s12871-026-03730-1.

## Introduction

Surgical decision-making is influenced by a multitude of factors, including disease characteristics, patient baseline conditions, financial considerations, and the availability of surgical expertise. Urological patients are often older and frail, making it crucial to prioritize life expectancy, comorbidity management, and organ function preservation when formulating surgical plans.

The advent of minimally invasive techniques, such as conventional laparoscopic surgery (CLS), has addressed many limitations of open surgery by minimizing incisions, thereby reducing postoperative pain, blood loss, and complication rates, while promoting faster recovery and shorter hospital stays [1–4]. Over the past decade, robotic-assisted laparoscopic surgery (RALS), with its enhanced ergonomics, has further expanded the scope of minimally invasive surgery, offering improved maneuverability in complex scenarios such as operations on obese patients [5, 6]. However, there is no clear consensus on whether RALS, CLS, or open surgery is superior, particularly regarding oncological outcomes. For example, an Italian study on prostate cancer suggested that RALS may be preferable for younger, healthier patients [7], while other reports indicate that RALS offers particular benefits in complex procedures for older patients or those with deep pelvic lesions [8–11]. Studies on radical prostatectomy have associated RALS with better short-term postoperative outcomes and potential long-term functional improvements compared to open or laparoscopic approaches. Conversely, other evidence suggests that for radical cystectomy, CLS may lead to fewer overall complications at 30 and 90 days postoperatively than RALS [12].

Both CLS and RALS require CO₂ pneumoperitoneum and Trendelenburg positioning, which impose physiological stress. Robotic procedures typically involve a steeper Trendelenburg angle, longer preparation and operative times, and thus may heighten perioperative risks for patients with poor cardiopulmonary reserve [13, 14]. Therefore, in addition to survival and complication rates, quality of life and functional recovery must be weighed in surgical planning. Thorough preoperative assessment is essential to identify patients capable of tolerating these physiological demands [15–17].

Postoperative recovery is a critical prognostic indicator, especially in patients with compromised baseline status, yet comparative studies on anesthesia-related recovery metrics between RALS and CLS remain limited. This study aims to compare postoperative recovery indicators between RALS and CLS in urological surgery from an anesthesiological standpoint, evaluating the relative advantages and disadvantages of each approach, and exploring the role of anesthesia in guiding surgical decision-making.

## Materials and methods

### Study population and eligibility criteria

This study has been approved by the Clinical Research Ethics Committee of the Second Hospital of Dalian Medical University Dalian China**(**KY2024-162-01) and conducted in accordance with the Declaration of Helsinki and the principles of the International Conference on Harmonization Good Clinical Practice Guideline.The requirement for obtaining written informed consent from participants was formally waived by the above ethics committee. This waiver was granted because this is a retrospective study that involved the analysis of existing, fully anonymized patient data, which presented no more than minimal risk to the participants.

A total of 831 patients aged 23–85 years who underwent RALS or CLS for urological conditions between 2021 and 2023 were initially enrolled.

Inclusion criteria were as follows: American Society of Anesthesiologists (ASA) physical status I–III; preoperative diagnosis of renal or prostate cancer; scheduled for partial nephrectomy or radical prostatectomy; absence of underlying systemic disease or presence of well-controlled comorbidities (hypertension, diabetes, or coronary heart disease with cardiac function class I–II); procedures performed by the same surgical and anesthesia team, with surgeons having undergone comparable robotic training.

Exclusion criteria included perioperative blood transfusion, postoperative transfer to the ICU, and incomplete medical records. After applying these criteria, 789 patients were included in the final analysis.

### Data collection and definitions

Demographic and clinical data were collected, including age, gender, BMI, and preoperative comorbidities. Outcome variables consisted of anesthesia time, surgical time, recovery time in PACU, resting and activity VAS scores, level of autonomous activity, and the incidence of nausea/vomiting, sore throat, anxiety, respiratory depression, dizziness and oxygen uptake rate on postoperative day 1 (POD1).

All data were extracted from electronic health records (EHR) and postoperative nursing reports. The following definitions were applied:

- Recovery time in PACU: duration of stay in the PACU.
- Pain intensity: assessed using a 10-point VAS, where 0 indicated no pain; 1–3, mild pain; 4–6, moderate pain affecting sleep; and 7–10, severe pain.
- Activity level: graded as 1 (fully independent), 2 (assisted or bedside activity), 3 (bedbound with movement), or 4 (completely immobile).
- Nausea/vomiting: classified as 0 (none), 1 (mild to moderate), or 2 (severe).

The authors acknowledge that several outcomes in this retrospective study were defined based on clinical documentation rather than validated instruments, which represents an inherent limitation. Specifically:

- Anxiety incidence was defined based on documentation in nursing or physician notes of terms such as “anxious,” “nervous,” or “worried” during the postoperative period, rather than a standardized anxiety scale.
- Dizziness was defined as patient-reported or nurse-observed complaints of vertigo, lightheadedness, or feeling faint, documented in postoperative records.
- Autonomic mobility grading was assessed by nursing staff based on observed patient activity, as defined in the grading criteria above. This assessment, while clinically practical, is inherently subjective and may be susceptible to inter-observer variability.

These pragmatic definitions reflect the constraints of retrospective data extraction from electronic health records and highlight the need for prospective studies using validated instruments to confirm these findings.

All patients received a standardized anesthetic protocol and uniform patient-controlled analgesia (PCA) pump settings for induction and maintenance of anesthesia.

### Statistical analysis

Statistical analyses were performed using R software (version 4.2.1). To mitigate selection bias, propensity score matching (PSM) was conducted in a 1:1 ratio using the nearest neighbor method with a caliper of 0.02. The propensity score was modeled based on clinically relevant covariates: age, gender, BMI, surgery type, ASA classification, and key comorbidities. Balance between the matched RALS and CLS groups was assessed using standardized mean differences (SMD < 0.1 indicated good balance). After matching, continuous and categorical variables were compared using independent samples t-tests and chi-square tests, respectively. A p-value < 0.05 was considered statistically significant.

## Results

### Propensity score matching

**Fig. 1:**
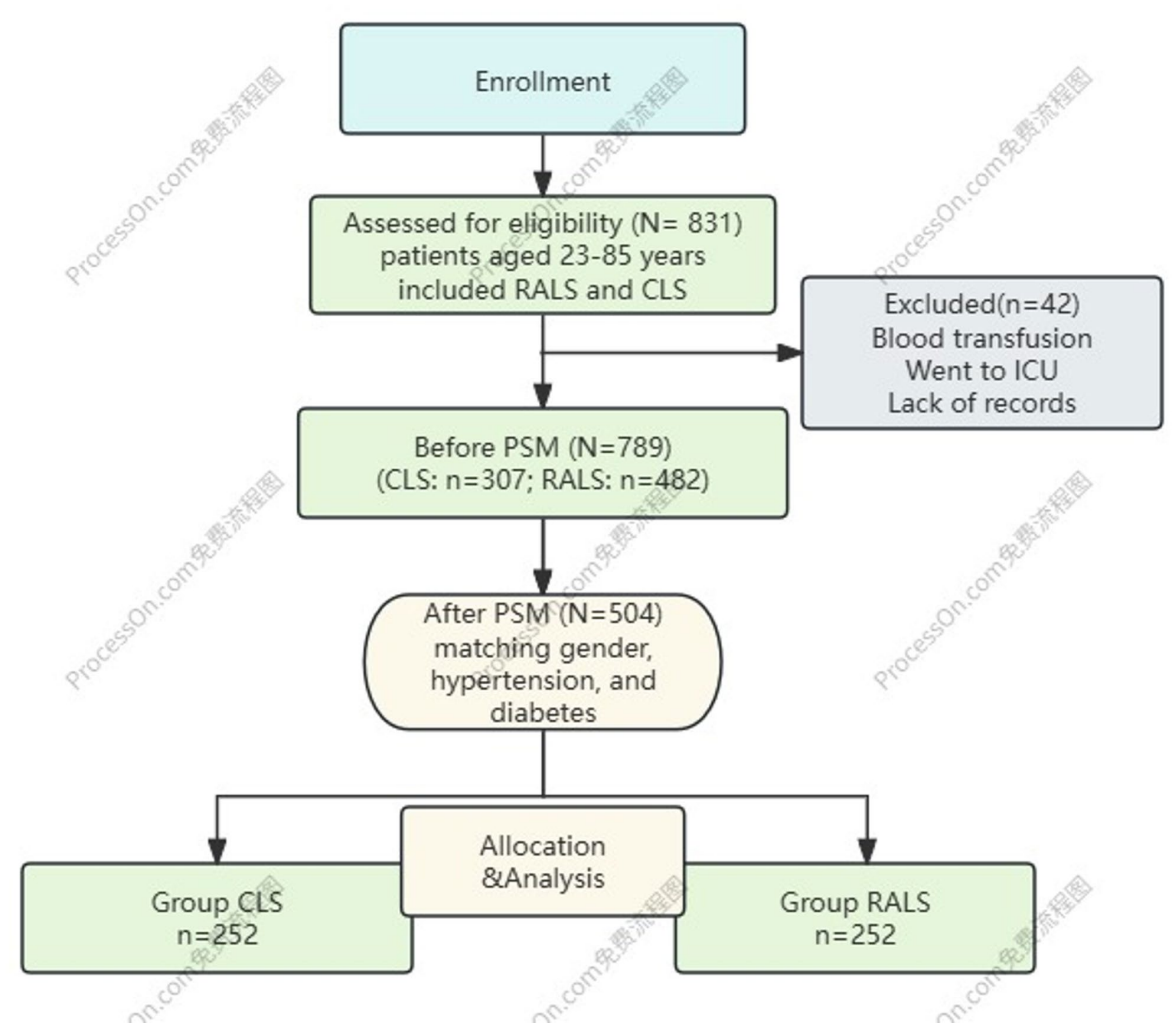
Flowchart of enrollment

PSM was successfully performed in a 1:1 ratio with a caliper width of 0.02. Before matching, the study included 789 patients (CLS: *n* = 307; RALS: *n* = 482), with significant baseline differences in gender, hypertension, and diabetes observed between the two groups. After PSM, 504 patients were matched, with 252 in each group. As summarized in flowchart Fig. 1; Table 1. After matching, all covariates achieved balance with standardized mean differences (SMDs) < 0.1, indicating good covariate balance between the two groups (SMDs for all covariates are presented in Supplementary Table 1).

**Table 1:** Baseline characteristics before and after propensity score matching

Variable	Before PSM			After PSM		
	CLS(*n* = 307)	RALS(*n* = 482)	*p*	CLS(*n* = 252)	RALS(*n* = 252)	*p*
Age	60.85 ± 12.07	60.62 ± 12.77	0.79	61.02 ± 12.22	59.77 ± 13.10	0.27
BMI	24.77 ± 3.16	25.22 ± 3.58	0.07	24.74 ± 2.96	24.72 ± 3.17	0.92
Cases and Percentage of>70y n(%)	91(29.7)	136(28.2)	0.18	82(28.8)	87(31.3)	0.26
Cases and Percentage of<70y n(%)	216(70.3)	346(71.8)	0.22	170(71.2)	165(68.8)	0.18
Gender n (%)						
Male	201(65.5)	350 (72.6)	0.03*	171 (67.9)	171 (67.9)	1.00
Female	106(34.5)	132 (27.4)		81 (32.1)	81 (32.1)	
Hypertension n (%)						
None	205 (66.8)	356 (73.9)	0.03^*^	175 (69.4)	179 (71.0)	0.77
Yes	102 (33.2)	126 (26.1)		77 (30.6)	73 (29.0)	
Diabetes n (%)						
None	257 (83.7)	429 (89.0)	0.04^*^	222 (88.1)	223 (88.5)	1.00
Yes	50 (16.3)	53 (11.0)		30 (11.9)	29 (11.5)	
Coronary heart disease n (%)						
None	291 (94.8)	454 (94.2)	0.84	239 (94.8)	242 (96.0)	0.67
Yes	16 (5.2)	28 (5.8)		13 (5.2)	10 (4.0)	
Percentage partial nephrectomy n (%)	203 (66.2)	338 (70.1)	0.02^*^	166 (65.9)	171(67.8)	0.89
Percentage radical prostatectomy n (%)	104 (33.8)	144 (29.9)	0.03^*^	86 (34.1)	81(32.2)	0.74

*BMI *Body Mass Index

*p < 0.05

### Comparison of postoperative outcomes after matching

Intergroup comparisons of postoperative outcomes were performed using independent samples t-tests for continuous variables and chi-square tests for categorical variables.

As shown in Table 2, the RALS group had significantly longer anesthesia time and operation time compared to the CLS group, along with a higher activity VAS score (all *P* < 0.05).

**Table 2:** The differences in recovery time and VAS between groups were assessed after matching

	CLS(*n* = 252)	RALS(*n* = 252)	*p*
Duration of anesthesia (min)	136.56 ± 48.35	158.52 ± 56.89	0.035*
Duration of operation (min)	125.34 ± 47.10	140.15 ± 55.21	0.002*
Duration of PACU (min)	26.51 ± 19.41	24.63 ± 20.35	0.337
Rest-VAS	1.06 ± 1.45	0.96 ± 1.20	0.403
Activity-VAS	1.86 ± 1.61	2.19 ± 1.58	0.047*

*PACU *Post-Anesthesia Care Unit, *Rest-VAS *Visual Analogue Scale during rest, *Activity-VAS *Visual Analogue Scale during the activity

* *p* < 0.05

Regarding postoperative recovery indicators (Table 3), the incidence of autonomous activity was significantly lower in the RALS group, whereas the incidence of nausea and vomiting was higher (all *P* < 0.05). Furthermore, as detailed in Table 4, dizziness was more frequent in the RALS group, while the incidence of anxiety was lower than in the CLS group (*P* < 0.05). No statistically significant differences were found between the groups for the remaining outcome measures (*P* > 0.05).

**Table 3:** Disparities in postoperative automatic activity levels and incidence of nausea and vomiting were observed between the two matched groups

	CLS(*n* = 252)	RALS(*n* = 252)	*p*
Activity status *n* (%)			
1.Autonomic mobility	73(29.1)	49 (19.5)	< 0.001**
2.Bedside or support mobility	18(7.3)	21(8.5)	0.327
3.Bed mobility	130(51.4)	153(60.6)	0.873
4.None mobility	31(12.2)	29(11.4)	0.763
Nausea & Vomiting n (%)			
1.None	182(72.1)	169(67.1)	0.043*
2.Mild & Moderate	70(27.9)	75(29.8)	0.651
3. Severe	0(0)	8(3.1)	0.026*

*p < 0.05

**Table 4:** The occurrence of other postoperative complications differed between groups after matching

	CLS(*n* = 252)	RALS(*n* = 252)	*p*
Postoperative oxygen uptake rate (%)	14(7.8)	10 (5.2)	0.739
Pharyngalgia incidence (%)	18(10.1)	12 (6.3)	0.12
Anxiety incidence (%)	30(16.8)	24 (12.6)	0.048*
Incidence of respiratory depression (%)	3 (1.7)	1(0.5)	0.144
Incidence of dizziness (%)	32(18)	47(25)	0.032*

*p < 0.05

## Discussion

The comparison of anesthesia recovery outcomes between RALS and CLS remains inconclusive. While a study on endometrial cancer reported no differences in complications or hospital stay—though operative time was longer in RALS, largely dependent on surgical experience [18]—our findings align with previous reports indicating that RALS entails longer preparation, operative, and anesthesia times compared to CLS [13–14]. Although we hypothesized that prolonged anesthesia might delay recovery, no significant difference was observed in PACU stay between the two groups, a result consistent with a prior randomized trial [19].

Notably, an earlier study suggested that recovery time is influenced by total anesthetic dose and surgical technique [18]. Emergence from anesthesia is generally affected by factors such as drug accumulation, blood loss, and CO₂ retention. In the present study, however, the use of short-acting anesthetics (remifentanil and propofol), deep neuromuscular blockade to accommodate higher pneumoperitoneum pressures, and reversal with sugammadex, along with a consistent perioperative team and protocol, likely minimized variations in anesthetic and surgical factors. Furthermore, propensity score matching balanced key baseline characteristics. Therefore, under standardized anesthetic management, no significant difference in PACU recovery time was detected between RALS and CLS.

While minimally invasive surgery is known to reduce postoperative analgesic use, accelerate bowel recovery, and shorten hospital stay [20, 21], comparative evidence on pain outcomes between RALS and CLS remains limited. Some studies suggest RALS offers superior pain control and faster recovery [22, 23], such as Tewari et al., who reported lower VAS scores on POD1 with RALS [24]. Conversely, Webster et al. found only early-phase differences in pain, with no significant variation by POD1 or POD14 [25]. In our study, although resting VAS scores were similar between groups under PCA, activity-induced pain was significantly higher in the RALS group. This may be explained by the larger number of trocar sites in RALS, which has been identified as a primary source of postoperative pain [26, 27], potentially also contributing to reduced early autonomous activity. Consistent with other parameters, a study by Boo-young et al. involving 57 prostatectomy patients found no advantage for RALS in blood loss, PACU time, or hospitalization; instead, longer operative time increased anesthetic and analgesic requirements [19].

An important consideration is whether the statistically significant differences observed in this study are clinically meaningful. The difference in activity-related VAS scores between groups was approximately 0.3 points on a 10-point scale. While the minimal clinically important difference (MCID) for postoperative pain VAS has not been firmly established, previous research suggests that a change of 1-1.5 points is typically required for patients to perceive a meaningful difference in pain intensity Reference. Similarly, the 22-minute difference in anesthesia time, while statistically significant, may not translate into clinically meaningful delays in recovery or discharge, particularly in the context of standardized ERAS protocols. These modest effect sizes suggest that the observed differences, while potentially indicative of underlying physiological processes, may not be readily perceptible to patients or significantly alter clinical recovery trajectories. This underscores the need for prospective studies designed to assess patient-centered outcomes and functional recovery beyond simple statistical comparisons.

The higher incidence of nausea and vomiting in the RALS group may be attributable to prolonged pneumoperitoneum and steep Trendelenburg positioning. Although previous studies have reported increased pharyngeal pain and laryngeal edema after RALS [28], we observed no such difference, possibly due to the use of deep neuromuscular blockade to reduce pharyngeal muscle tension and mucosal edema. The absence of between-group differences in supplemental oxygen requirement or respiratory depression suggests effective reversal of anesthesia and neuromuscular blockade. The lower incidence of postoperative anxiety observed in the RALS group is intriguing but requires cautious interpretation. This finding may reflect several factors beyond a direct effect of the surgical approach itself. Patients undergoing robotic surgery often receive more detailed preoperative counseling about the advanced technology, which may alleviate anxiety through enhanced expectations or perceived access to cutting-edge care. Alternatively, patients who opt for or are selected for robotic surgery may differ in unmeasured psychosocial characteristics, such as health literacy or optimism. Additionally, the novelty and perceived prestige of robotic technology may itself exert a placebo-like effect on anxiety. Given the retrospective nature of this study and the lack of validated anxiety scales, these explanations remain speculative. Prospective studies incorporating preoperative psychological assessments and patient expectation surveys are needed to elucidate the mechanisms underlying this observation, whereas increased dizziness may relate to elevated intracranial pressure from prolonged steep positioning.

This study provides an anesthesiologist-centered evaluation of RALS versus CLS in urological surgery. RALS was not superior across all anesthesia-related recovery metrics. These findings suggest that surgical approach selection may benefit from incorporating consideration of the patient’s physiological reserve. The pronounced physiological stresses imposed by RALS—prolonged pneumoperitoneum and extreme Trendelenburg position—may pose greater challenges for patients with cardiopulmonary compromise [29], though this hypothesis requires direct investigation in prospective studies. Additionally, the physical bulk of robotic equipment has been suggested as a potential consideration for emergency access [30], though our study did not evaluate this factor.

This study is among the few to analyze differences in postoperative recovery between robotic and laparoscopic surgeries from an anesthesiological perspective. Although retrospective in design, the use of PSM effectively balanced key confounding variables. Several limitations should be acknowledged. First, despite the use of propensity score matching to balance observed covariates, this retrospective single-center study is subject to inherent biases and potential residual confounding. Important variables that could influence perioperative outcomes were not included in the propensity model, including surgeon experience over time (learning curve effects), subtle differences in anesthetic drug dosing or administration timing, intraoperative events not captured in electronic records (e.g., hemodynamic instability, desaturation episodes), and patient positioning specifics such as operative position angle and duration of pneumoperitoneum. Second, the sample size was reduced after matching, which may limit statistical power for detecting smaller differences in outcomes. Third, this study focused primarily on anesthesia-related recovery indicators and did not evaluate several important surgical outcomes, including intraoperative blood loss, warm ischemia time, conversion to open surgery, major complications, length of stay, or total cost. Trends in perioperative cardiopulmonary function were also not analyzed. These aspects warrant further investigation in prospective, randomized controlled trials designed to capture both surgical and anesthesia-related outcomes comprehensively. Additionally, while propensity score matching included surgery type as a covariate, the decision to pool partial nephrectomy and radical prostatectomy patients warrants discussion. These procedures differ in operative complexity, patient positioning (e.g., flank versus lithotomy/Trendelenburg), and physiological stress profiles. Although matching on surgery type helps mitigate these differences, residual confounding related to procedure-specific factors cannot be fully excluded. Future studies with larger sample sizes should consider stratified analyses by procedure type to validate these findings.

In conclusion, this propensity score-matched analysis from the anesthesiologist’s perspective suggests that RALS is associated with longer anesthesia duration, higher activity-related pain, and increased rates of nausea/vomiting and dizziness compared to CLS. These observations raise the hypothesis that the choice of surgical approach may benefit from considering patient-specific factors such as cardiopulmonary reserve and tolerance for prolonged steep Trendelenburg positioning. However, given the retrospective design and modest effect sizes, these findings should be considered exploratory and require confirmation in prospective, randomized controlled trials. The potential role of anesthesiologists in preoperative decision-making within a Multidisciplinary Team (MDT) framework warrants further investigation.

## Supplementary Information

Supplementary Material 1.

## Data Availability

The datasets used and/or analyzed during the current study are available from the corresponding author on reasonable request.

## Abbreviations

RALS: Robotic-assisted laparoscopic surgery
CLS: Conventional laparoscopic surgery
ERAS: Enhanced Recovery After Surgery
PSM: Propensity score matching
PACU: Post-anesthesia care unit
VAS: Visual analogue scale
POD1: Postoperative day 1
BMI: Body mass index
CO^2^: Carbon dioxide
ASA: American Society of Anesthesiologists
ICU: Intensive care unit
PCA: Patient-controlled analgesia
MDT: Multidisciplinary Team
