# Supplementary material for: The anesthesiologist’s perspective in choosing between robotic-assisted and conventional laparoscopic urological surgery: a propensity score-matched analysis
Source: BMC Anesthesiol. 2026 Mar 31;26:289. doi: 10.1186/s12871-026-03730-1 (PMC13159358; doi:10.1186/s12871-026-03730-1)
Supplement: Supplementary file 1 — Supplementary Material 1. [file 12871_2026_3730_MOESM1_ESM.docx]

Supplementary Table 1. Standardized Mean Differences After Propensity Score Matching

| Variable | Type | SMD after matching |
| --- | --- | --- |
| Age | Continuous | 0.099 |
| BMI | Continuous | 0.007 |
| Gender | Categorical | 0.000 |
| Hypertension | Categorical | 0.035 |
| Diabetes | Categorical | 0.012 |
| Coronary heart disease | Categorical | 0.058 |
| Surgery type | Categorical | 0.041 |

Note: All SMDs < 0.1 indicate excellent covariate balance after matching.
